# Dapagliflozin: a sodium–glucose cotransporter 2 inhibitor, attenuates angiotensin II-induced cardiac fibrotic remodeling by regulating TGFβ1/Smad signaling

**DOI:** 10.1186/s12933-021-01312-8

**Published:** 2021-06-11

**Authors:** Yuze Zhang, Xiaoyan Lin, Yong Chu, Xiaoming Chen, Heng Du, Hailin Zhang, Changsheng Xu, Hong Xie, Qinyun Ruan, Jinxiu Lin, Jie Liu, Jinzhang Zeng, Ke Ma, Dajun Chai

**Affiliations:** 1 Cardiovascular Department, The First Affiliated Hospital, Fujian Medical University, Fujian Institute of Hypertension, 20 Chazhong Road, Fuzhou, 350005 China; 2 Echocardiological Department, The First Affiliated Hospital, Fujian Medical University, Fuzhou, 350005 China; 3 Editorial Department of Chinese Journal of Hypertension, Fuzhou, 350005 China; 4 School of Pharmacy College, Xiamen University, Xiamen, 361102 China; 5 Clinical Research Center, The First Affiliated Hospital, Fujian Medical University, 20 Chazhong Road, Fuzhou, 350005 China

**Keywords:** Sodium–glucose cotransporter 2 inhibitors, Dapagliflozin, Angiotensin II, Cardiac fibrotic remodeling, TGFβ1/Smad signaling

## Abstract

**Background:**

Cardiac remodeling is one of the major risk factors for heart failure. In patients with type 2 diabetes, sodium–glucose cotransporter 2 (SGLT2) inhibitors reduce the risk of the first hospitalization for heart failure, possibly through glucose-independent mechanisms in part, but the underlying mechanisms remain largely unknown. This study aimed to shed light on the efficacy of dapagliflozin in reducing cardiac remodeling and potential mechanisms.

**Methods:**

Sprague–Dawley (SD) rats, induced by chronic infusion of Angiotensin II (Ang II) at a dose of 520 ng/kg per minute for 4 weeks with ALZET® mini-osmotic pumps, were treated with either SGLT2 inhibitor dapagliflozin (DAPA) or vehicle alone. Echocardiography was performed to determine cardiac structure and function. Cardiac fibroblasts (CFs) were treated with Ang II (1 μM) with or without the indicated concentration (0.5, 1, 10 μM) of DAPA. The protein levels of collagen and TGF-β1/Smad signaling were measured along with body weight, and blood biochemical indexes.

**Results:**

DAPA pretreatment resulted in the amelioration of left ventricular dysfunction in Ang II-infused SD rats without affecting blood glucose and blood pressure. Myocardial hypertrophy, fibrosis and increased collagen synthesis caused by Ang II infusion were significantly inhibited by DAPA pretreatment. In vitro, DAPA inhibit the Ang II-induced collagen production of CFs. Immunoblot with heart tissue homogenates from chronic Ang II-infused rats revealed that DAPA inhibited the activation of TGF-β1/Smads signaling.

**Conclusion:**

DAPA ameliorates Ang II-induced cardiac remodeling by regulating the TGF-β1/Smad signaling in a non-glucose-lowering dependent manner.

**Supplementary Information:**

The online version contains supplementary material available at 10.1186/s12933-021-01312-8.

## Introduction

Accentuated deposition of extracellular matrix (ECM) proteins contributed from cardiac fibroblasts (CFs) activation is a key feature of pathological myocardial remodeling involved in nearly all etiologies of heart diseases, which increases myocardial stiffness and ultimately leads to the progression of heart failure [1]. In the myocardium's fibrotic remodeling, activated CFs typically undergo myofibroblast transdifferentiation, expressing contractile proteins, such as α-smooth muscle actin (α-SMA), and synthesizing large amounts of structural ECM proteins mainly including types I and type III fibrillar collagen [2, 3]. Several mediators promote the development of cardiac fibrosis regardless of the underlying pathology and initiate factors. Angiotensin II (Ang II), the major effector in the renin–angiotensin–aldosterone system (RAAS), serves as a potent activating stimulus for cardiac fibrosis and cardiomyocyte hypertrophy [4]. The model of chronic subjection to Ang II in murine is widely used to mimic chronic hypertension upon neurohumoral activation, where reactive interstitial and perivascular fibrosis were observed [5]. Among a wide range of fibrogenic signaling pathways, transforming growth factor-β1 (TGF-β1)/Smad is crucial for the induction and maintenance of CFs activation and collagen synthesis, which partially mediates Ang II-induced structural remodeling [6]. Although extensive studies and interventions have been carried out to inhibite cardiac fibrosis and reverse myocardial remodeling, there is still a clinical desire for novel pharmacological approaches [7]. In recent years, studies have revealed that sodium–glucose cotransporter 2 inhibitors (SGLT2i), a new class of anti-diabetic drugs, could play a cardioprotective role beyond the glucose-lowering effect [8]. In Dapagliflozin in Patients with Heart Failure and Reduced Ejection Fraction (DAPA-HF) trial, among patients with heart failure and a reduced ejection fraction, dapagliflozin (DAPA) reduced the risk of worsening heart failure or cardiovascular death, regardless of the presence or absence of diabetes [9]. In rats with cardiac ischemia/reperfusion injury, DAPA pretreatment could provide cardioprotective effects by reducing the infarct size [10]. Empagliflozin (EMPA; another SGLT2i) has been reported to improve LV ejection fraction and cardiac remodeling associated with improvements in cardiac metabolism and cardiac ATP production in myocardial infarction rats models [11].

Furthermore, experiment with human cardiac myofibroblast and found that EMPA can attenuate TGFβ1-induced fibroblast activation and cell-mediated collagen remodeling [12]. In general, there are relatively fewer studies concerning the cardioprotective potential of SGLT2i on patients with non-diabetic cardiovascular disease. And more histopathological evidence from animal models is needed to prove the effect of SGLT2i in alleviating cardiac fibrotic remodeling. However, the possible mechanisms by which SGLT2i achieve cardiovascular benefits remain to be elucidated since some studies believe that SGLT2 is not expressed in the human heart [13].

Therefore, we performed the present study to confirm the glycemic-control-independent effect of DAPA towards cardiac remodeling using rats with chronic Ang II perfusion and explore the regulatory signaling with a focus on the TGF-β pathway. Our data suggested that DAPA may help prevent patients from cardiac dysfunction without diabetes and revealed possible mechanisms of the cardioprotective effects of SGLT2i.

## Materials and methods

### Modeling and grouping

Eight-week-old male Sprague–Dawley (SD) rats (180 ± 20 g) purchased from Shanghai Laboratory Animal Center. All animals were housed in a room under temperature control at 23 °C and kept on a 12 h light/dark cycle. Commercial chow and water were supplied ad libitum. The animal experiment was completed in the Central Laboratory of the First Affiliated Hospital of Fujian Medical University. Twenty-four rats were randomly allocated into four groups (n = 6 for each group): Control saline infusion group (CTL); Control treated with dapagliflozin group (5 mg/kg/day) [14] (CTL + DAPA); Ang II infusion with vehicle (0.9% sterile saline) gavage group (Ang II); Ang II infusion with DAPA gavage group (Ang II + DAPA). Ang II (520 ng/kg/min, A9525, Sigma-Aldrich, St Louis, MO) or saline was continuously infused into rats through the osmotic pump (ALZET® Osmotic Pumps 2006, DURECT) for 4 weeks [15]. We administered the DAPA (gift of Astra Zeneca) or 0.9% sterile saline by gastric gavage continued up to 4 weeks. At the end of the treatment, blood pressure (BP) and body weight were measured, and then rats were anesthetized, echocardiography was performed to determine cardiac structure and function, blood samples and the heart were collected.

### BP measurement

After completing 4-week drug administrations, BP was measured using a non-invasive tail-cuff system (Softron BP-2010A, Beijing Biotechnology Co., Ltd., Beijing, China). Conscious rats were acclimated to the apparatus for 5 min before starting measurement, and then placed on the warmed platform of the machine (37 °C). The BP data were collected three times under a resting state, and the average value was calculated.

### Echocardiography

Four weeks after drug administrations, rats were anesthetized with 2% isoflurane via face mask. Transthoracic echocardiography (Vivid E95, GE Health-care, Horten, Norway) was performed using a 12S probe at frequencies of 4–12 MHz. M-mode images were obtained from parasternal short axis view of the left ventricular- papillary muscle levels to analyze the cardiac structure and function. Pulsed wave Doppler blood flow images of the apical four-chamber view at the mitral level and tissue Doppler images of the lateral and septal mitral annulus were recorded. Heart rate (HR) were recorded by synchronized electrocardiography. Images were quantified and analyzed by the Echopack 113.1.3 image analysis system (GE Healthcare). End-diastolic interventricular septum thickness (IVSd), end-diastolic left ventricular posterior wall thickness (LVPWd), and end-diastolic and end-systolic left ventricular internal diameters (LVIDd and LVIDs) were measured, and the left ventricular fractional shortening (LVFS) and the left ventricular ejection fraction (LVEF) were calculated as described previously [16].

The software module for quantitative tissue velocity imaging (QTVI) (GE Healthcare) was utilized to measure the systolic peak velocities (s, s′), early diastolic peak velocities (e, e′) and end-diastolic peak velocities (a, a′) of the lateral and septal mitral annulus on the apical four-chamber view. The systolic average peak velocity (S_ave_), early diastolic average peak velocity (e_ave_), late diastolic average peak velocity (a_ave_) and the ratio of E/e_ave_ of the lateral and septal mitral annulus were calculated.

The 2D-strain tool of the Q-analysis software (GE Healthcare) was utilized to analyze seven tracking points located at the apex, the lateral and septal basal segments, the medium segment, and apical segment boundaries were selected sequentially on the left ventricular endocardial surface on the apical four-chamber view section image. Sampling frames were automatically generated, and the sizes of the frames were adjusted for consistency with ventricular wall thickness. The global longitudinal strain (GLS) was calculated. All measurements were performed by an echocardiologist unaware of the identities of the experimental groups, and the values of the above parameters were calculated from the average of five continuous cardiac cycles.

### Measurements of laboratory chemistry and circulating bioactive peptides

After echocardiography was performed, blood samples were collected from the abdominal aorta and centrifuged to isolate serum and stored at − 80 °C until assay. Glucose, lipid (Glu), blood urea nitrogen (BUN), serum creatinine (SCr), serum alanine aminotransferase (ALT), aspartate aminotransferase (AST), lactate dehydrogenase (LDH), creatine kinase (CK), and CK-MB were determined by the colorimetric method utilizing an automatic biochemical analyzer (Roche, Shanghai, China).

Circulating vasoactive peptide concentrations were determined using Enzyme-linked Immunosorbent Assay (ELISA) kits according to the manufacturer’s protocol. The serum levels of atrial natriuretic peptide (ANP, Uscnk, Wuhan, China, Cat no: CEA225Ra), brain natriuretic peptide (BNP, Uscnk, Wuhan, China, Cat no: CSB-E07972r), Ang II (Cusabio Biotech, Wuhan, China, Cat no: CEA005Ra), and insulin (INS, Uscnk, Wuhan, China, Cat no: CEA448Ra) were measured. All assays were performed in duplicate.

### Histopathology and immunohistochemistry

After blood samples were collected, the heart was harvested, and the LV were separated, and weighed. The heart weight to tibial length ratio (HW/TL, g/cm) and the LV weight to tibia length (LVW/TL, g/cm) ratio were calculated.

The heart tissue was fixed in 10% formalin for 24 h, dehydrated, embedded in paraffin, and sectioned at 5 μm thickness, and these sections were mounted on glass slides. Hematoxylin–Eosin (H&E, Sigma-Aldrich, MO, USA) staining and Picric Acid-Sirius Red staining were used for histological and fibrosis analysis respectively.

For immunohistochemical staining, primary antibodies against Collagen I (Abcam, Cat no: ab34710), Collagen III (Abcam, Cat no: ab32854), α-smooth muscle actin (α-SMA) (Cell Signaling Technology, Cat no: #19245), TGF-β1 (ABclonal, Cat no: A2124) were used. Immunohistochemistry of cardiac sections was performed using a horseradish peroxidase-3′-3′-diaminobenzidine kit (Zhongshan Jinqiao Biotechnology, Beijing, China, Cat no: PV-9000) following manufacturers’ instructions. To quantify cardiomyocyte area and fibrotic percentage, the heart sections’ images were obtained using an optical microscope and then analyzed using Image J version 1.8.0 (National Institutes of Health, USA). Three visual fields were randomly selected for each heart tissue section, and their average values were taken as data for each rat.

### Cell culture and treatment

Primary cardiac fibroblasts (CFs) isolated from adult rat ventricular tissues as previously described [16]. Briefly, cells were grown in M199 medium (HyClone, Cat no: SH30253.01) (10% fetal bovine serum and 1% penicillin/streptomycin) at 5% CO_2_, 37 °C incubator. The purity of CFs in the culture was higher than 95% and only passages from 1 to 3 were used in the study to avoid age-dependent culture modifications. After incubation in a 0.5% fetal bovine serum M199 medium for 12 h, CFs were treated 24 h with of Ang II (1 μM) with or without DAPA at a concentration of 0.5, 1, 10 μM. (MedChemExpress, Cat no: HY-10450).

### Immunoblot analyses

Samples were homogenized in lysis buffer (50 mM Tris–HCl, pH 7.4, 1 mM EDTA, 100 mM NaCl, 20 mM NaF, 3 mM Na_3_VO_4_, 1 mM PMSF, and protease inhibitor cocktail). Heart tissues and cell lysates were separated by 8–10% SDS-PAGE and transferred to nitrocellulose membranes. Antibodies used were rabbit polyclonal antibody against Collagen I (Abcam, Cat no: ab34710), rabbit polyclonal antibody against Collagen III (Abcam, Cat no: ab32854), rabbit monoclonal antibodies against β-actin (Santa Cruz Biotechnology, Cat no: sc-47778), α-smooth muscle actin (α-SMA) (Cell Signaling Technology, Cat no: # 19245), Smad2 (Cell Signaling Technology, Cat no: #5339), Phospho-Smad2 (Cell Signaling Technology, Cat no: #18338), Smad3 (Sigma, Cat no: AV100621), Phospho-Smad3 (Sigma, Cat no: SAB4504210), Smad7 (Abcam, Cat no: ab216428) and rabbit polyclonal antibody against TGF-β1 (ABclonal, Cat no: A2124) at 4 °C overnight. The integrated density value of the target protein bands was measured by Image J software. β-actin was used to normalize the target protein level on the same blot.

### Statistical analysis

All results are expressed as the means ± SD, and all comparisons were analyzed using one-way ANOVA followed by the Student–Newman–Keuls test for multiple comparisons using IBM SPSS statistics 22 software. Results were considered significant when a value of *P* < 0.05 and values *P* < 0.01 were considered highly significant.

## Results

### Effects of DAPA on biochemical indicators and BP in rats

As shown in Table 1, there was no significant difference in the plasma glucose levels and insulin concentrations among the four groups throughout the study, indicating that Ang II-infused rat model was free of diabetes. Compared to the CTL group, chronic perfusion of Ang II significantly increased SBP and DBP. DAPA intervention showed a certain BP-lowering effect, but there was no significant difference between Ang II-infusion and Ang II + DAPA group (Table 2 and Additional file 1: Table S1). Chronic Ang II perfusion significantly decreased the body weight of rats after 4 weeks. At the end of the the experiment, there were no difference in total serum cholesterol (TC), triglyceride (TG), and low-density lipoprotein (LDL), and we did not find any liver, skeletal muscle, or kidney toxicity related to DAPA treatment (Table 1).

**Table 1:** Serum and biochemical indicators of Ang II rats treated with vehicle or DAPA for 4 weeks

	CTL	CTL + DAPA	Ang II	Ang II + DAPA
Glu (mM)	8.29 ± 1.29	8.33 ± 1.28	8.09 ± 1.12	8.05 ± 1.17
INS (pg/ml)	223 ± 38.94	224.53 ± 66.16	205.95 ± 49.33	215.59 ± 43.51
TC (mM)	1.8 ± 0.2	1.6 ± 0.37	1.98 ± 0.48	1.85 ± 0.27
TG (mM)	1.14 ± 0.49	1.08 ± 0.46	1.31 ± 0.46	1.22 ± 0.56
LDL-C (mM)	0.35 ± 0.1	0.36 ± 0.09	0.35 ± 0.12	0.38 ± 0.07
HDL-C (mM)	0.53 ± 0.16	0.57 ± 0.05	0.51 ± 0.19	0.59 ± 0.1
ALT (U/I)	53.67 ± 8.59	61 ± 14.3	59.5 ± 7.61	52.33 ± 6.62
AST (U/I)	244.09 ± 10.05	246.16 ± 38.39	229.17 ± 17.41	236 ± 12.18
CK (U/I)	331.14 ± 11.81	327.08 ± 15.24	317.88 ± 18.12	321.81 ± 15.85
CK-MB (U/I)	338.28 ± 13.79	337.41 ± 15.76	318.38 ± 20.62	326.69 ± 16.81
LDH (U/I)	360.22 ± 13.26	359.74 ± 13.15	340.61 ± 24.75	354.96 ± 14.97
BUN (mM)	6.61 ± 1.43	6.88 ± 1.76	7.84 ± 2.13	8.64 ± 2.05
SCr (μmol/l)	18.12 ± 3.32	19.72 ± 2.89	20.05 ± 4.59	18.48 ± 2.59
UA (μmol/l)	11.3 ± 7.12	13.38 ± 5.86	8.22 ± 3.5	7.57 ± 3.95

Values are presented as mean ± SD (n = 6 rats per group)

Every parameter showed no significant difference among the four groups. Data are not significant for each group

*Glu* glucose, *INS* insulin, *TC* total serum cholesterol, *TG* triglyceride, *LDL* low-density lipoprotein, *HDL* high-density lipoprotein, *SCr* serum creatinine, *ALT* serum alanine aminotransferase, *AST* aspartate aminotransferase, *LDH* lactate dehydrogenase, *CK* creatine kinase

**Table 2:** Physical and conventional echocardiographic parameters in normal and Ang II-infused rats treated with vehicle or DAPA

Parameters	CTL	CTL + DAPA	Ang II	Ang II + DAPA
BW (g)	362.17 ± 12.67	358 ± 9.93	320.33 ± 15.31*	318.5 ± 12.29*
SBP (mmHg)	131.95 ± 13.49	134.4 ± 16.43	228.28 ± 14.62*	222.67 ± 19.34*
DBP (mmHg)	102.6 ± 16.62	106.77 ± 11.2	190.02 ± 18.49*	173.1 ± 17.58*
HW (g)	1.0 ± 0.08	1.03 ± 0.07	1.33 ± 0.05*	1.14 ± 0.1*^†^
LVW (g)	0.71 ± 0.07	0.71 ± 0.08	1.0 ± 0.03*	0.85 ± 0.07*^†^
IVSd (mm)	1.83 ± 0.08	1.82 ± 0.06	2.5 ± 0.14*	1.85 ± 0.09^†^
LVEDd (mm)	7.45 ± 0.65	7.49 ± 0.19	6.28 ± 0.42*	7.06 ± 0.71^†^
LVEDs (mm)	4.49 ± 0.26	4.37 ± 0.23	3.37 ± 0.23*	4.0 ± 0.5*^†^
LVPWd (mm)	1.68 ± 0.1	1.74 ± 0.13	2.32 ± 0.13*	1.73 ± 0.12^†^
LVEDV (ml)	0.94 ± 0.22	0.92 ± 0.05	0.58 ± 0.1*	0.81 ± 0.24^†^
LVESV (ml)	0.22 ± 0.04	0.2 ± 0.02	0.1 ± 0.02*	0.17 ± 0.07*^†^
LVEF (%)	75.47 ± 3.21	78.02 ± 2.57	82.88 ± 0.79*	79.9 ± 1.99*^†^
LVFS (%)	39.51 ± 2.83	40.61 ± 1.41	46.32 ± 0.85*	43.46 ± 1.79*^†^
HR	389.48 ± 21.59	377.53 ± 17.21	409.01 ± 26.04	409.32 ± 21.06

Values are presented as mean ± SD (n = 6 rats per group)

Data are expressed as the mean ± SD

*BW* body weight, *SBP* systolic blood pressure, *DBP* diastolic blood pressure, *HW* heart weight, *LVW* left ventricle weight, *TL* tibia length, *IVSd* end-diastole interventricular septum thickness, *LVEDd* left ventricular end-diastolic dimension, *LVEDs* left ventricular end-systolic dimension, *LVEDV* left ventricular end-diastolic volume, *LVESV* left ventricular end-systolic volume, *LVPWd* diastolic left ventricular posterior wall thickness, *LVFS* left ventricular fractional shortening, *LVEF* the left ventricular ejection fraction, *HR* heart rate

*P < 0.05 relative to CTL group

^†^*P* < 0.05 relative to Ang II group

### DAPA attenuated cardiac remodeling and improved cardiac dysfunction induced by Ang II in rats

**Fig. 1:**
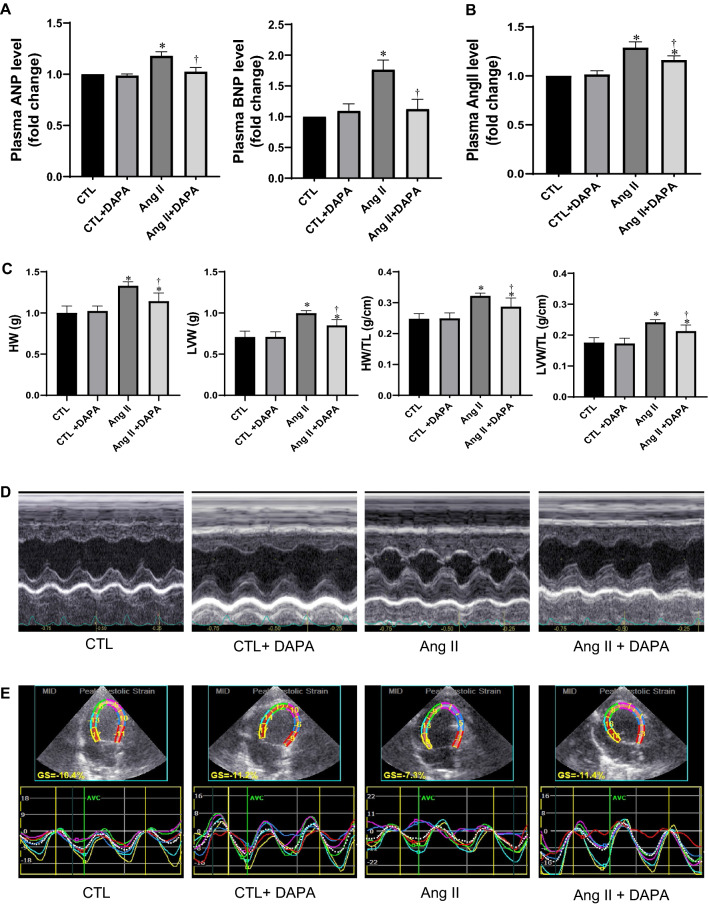
Administration of DAPA attenuates cardiac remodeling and improves cardiac dysfunction induced by Ang II in rats. **A**, **B** Analysis of ELISA of plasma ANP, BNP and Ang II levels; **C** HW, LVW, HW/TL ratios and HW/TL ratios; **D** representative examples of M-mode echocardiography images; **E** representative examples of left ventricular four-chamber 2Dspeckle tracking echocardiography imaging. Data are expressed as the mean ± SD (n = 6 rats per group). *P < 0.05 relative to CTL group. ^†^*P* < 0.05 relative to Ang II group

Chronic AngII infusion induced significant myocardial hypertrophy in rats. As shown in Fig. 1C, Ang II infused rats showed increases in HW, HW/TL, LVW, and LVW/TL ratios (*P* < 0.01), and these increases were significantly attenuated by DAPA (*P* < 0.05). In addition, we assessed the serum levels of ANP, BNP and Ang II using ELISA. The results showed that ANP, BNP, and Ang II were increased in Ang II group. DAPA treatment markedly attenuated these parameters to some extent (Fig. 1A, B).

To assess the effects of DAPA on the cardiac structure and function in Ang-II-infused rats, echocardiography was performed. As shown in Table 2 and Fig. 1D, after Ang II infusion, LVEF and LVFS were significantly increased compared to control group (82.88 ± 0.79% vs. 78.02 ± 2.57% and 46.32 ± 0.85% vs. 40.61 ± 1.41% respectively, *P* < 0.01). In Ang II + DAPA group, LVEF and LVFS were significantly decreased compared to the Ang II group (79.9 ± 1.99% vs. 82.88 ± 0.79% and 43.46 ± 1.79% vs. 46.32 ± 0.85% respectively, *P* < 0.05). IVSd and LVPWd in Ang II-infused group were significantly increased than those in CTL group, and the LVEDd, LVEDs, end-diastolic volume (EDV), end-systolic volume (ESV) of Ang II-infused rats were significantly lower than those in CTL group. These are consistent with previous reports [17, 18]. After DAPA treatment, these indicators of Ang-II-infused rats were sigfinicantly ameliorated (Table 2).

QTVI and 2D-speckle tracking echocardiography have higher sensitivities in detecting cardiac function compared to conventional echocardiography. Therefore, we assessed early cardiac function changes in rats and the protective effects of DAPA using this technique. Interestingly, we found that compared with the control group, s, s′, s_ave_, e, e′, e_ave_, a, a′ and a_ave_ in Ang II group were all significantly decreased (*P* < 0.05). Interestingly, DAPA pretreatment significantly increased s, s′, s_ave_, e, e′, e_ave_, a, a′ and a_ave_ of Ang II-infused rats (*P* < 0.05), e and e_ave_ in Ang II + DAPA group were significantly higher than those in Ang II group. Furthermore, E/e_ave_ ratios in Ang II group were higher than those in control group (Table 3). These suggest that DAPA can attenuate cardiac remodeling and left ventricular dysfunction induced by Ang II infusion in rats. As shown in Fig. 1E and Table 3, analysis of the 2D-speckle tracking echocardiographic images showed that compared with the control group, GLS of the left ventricle were all significantly lower in Ang II groups (*P* < 0.05). DAPA treatment increased these parameters in Ang II treated rats (*P* < 0.05).

**Table 3:** The effects of DAPA treatment on quantitative tissue velocity imaging parameters parameters

Parameters	CTL	CTL + DAPA	Ang II	Ang II + DAPA
s (mm/s)	2.68 ± 0.34	2.62 ± 0.28	1.66 ± 0.14*	1.96 ± 0.15^†^
a (mm/s)	1.87 ± 0.26	1.84 ± 0.22	1.38 ± 0.20*	1.66 ± 0.17^†^
e (mm/s)	2.89 ± 0.36	2.92 ± 0.44	1.84 ± 0.32*	2.66 ± 0.25^†^
a/e ratio	0.64 ± 0.12	0.63 ± 0.14	1.09 ± 0.13*	0.89 ± 0.11^†^
s′ (mm/s)	2.64 ± 0.31	2.60 ± 0.22	1.58 ± 0.28*	2.09 ± 0.29^†^
a′(mm/s)	2.42 ± 0.23	2.46 ± 0.28	1.86 ± 0.23*	2.27 ± 0.21^†^
e′ (mm/s)	3.74 ± 0.33	3.80 ± 0.42	2.21 ± 0.28*	2.86 ± 0.37^†^
a′/e′ ratio	0.82 ± 0.12	0.80 ± 0.14	0.96 ± 0.10*	0.88 ± 0.08^†^
s_ave_ (mm/s)	2.92 ± 0.24	2.89 ± 0.28	1.67 ± 0.23*	2.19 ± 0.18^†^
a_ave_ (mm/s)	2.57 ± 0.60	2.47 ± 0.52	1.87 ± 0.36*	2.19 ± 0.34^†^
e_ave_ (mm/s)	3.67 ± 0.32	3.68 ± 0.28	1.92 ± 0.32*	2.64 ± 0.24^†^
a_ave_/e_ave_	0.69 ± 0.14	0.68 ± 0.21	0.96 ± 0.21*	0.74 ± 0.27^†^
E/e_ave_	28.68 ± 0.46	29.64 ± 0.48	46.24 ± 0.58*	36.49 ± 0.54^†^
GLS (%)	− 12.18 ± 2.17	− 11.95 ± 2.42	− 7.57 ± 1.29*	− 12.64 ± 1.3^†^

Values are presented as mean ± SD (n = 6 rats per group)

s: peak systolic mitral annular velocity at lateral side; s′: peak systolic mitral annular velocity at septal side; s_ave_: average value of the peak systolic mitral annular velocity; a: late diastolic mitral annular velocity at lateral side; a′: late diastolic mitral annular velocity at septal side; a_ave_: average value of the late diastolic mitral annular velocity; e: early diastolic mitral annular velocity at the lateral side; *e*′: early diastolic mitral annular velocity at the septal side; e_ave_: average value of the early diastolic mitral annular velocity; E: Peak velocities of diastolic early transmitral Doppler flow; GLS: global longitudinal strain

*P < 0.05 relative to CTL group

^†^*P* < 0.05 relative to Ang II group

### DAPA attenuates Ang II-induced cardiac hypertrophy

**Fig. 2:**
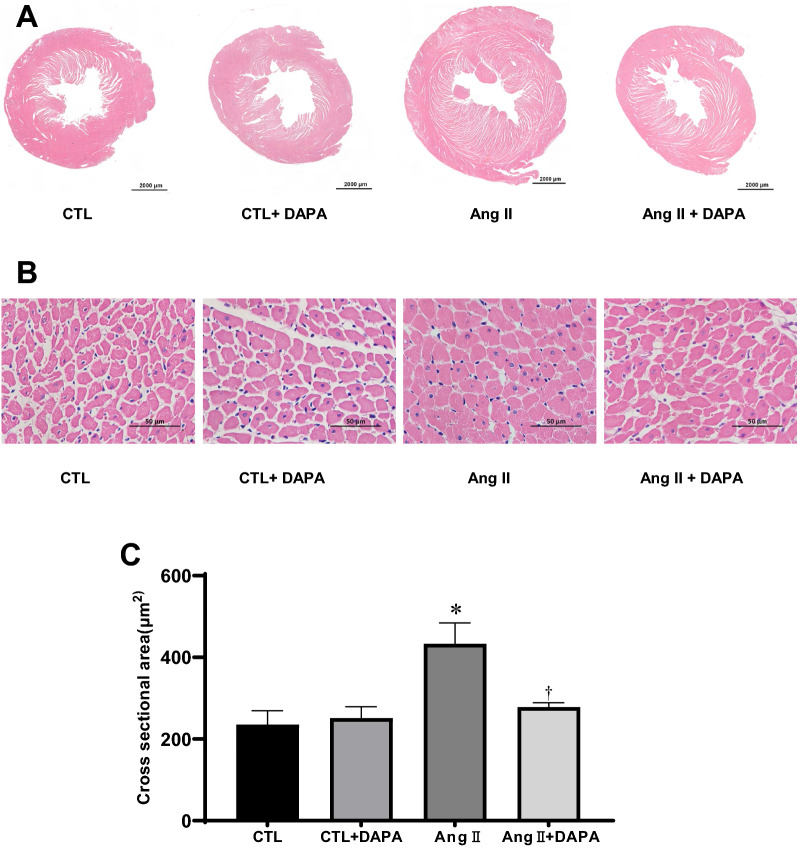
Administration of DAPA attenuates Ang II-induced cardiac hypertrophy. **A** H&E staining of the cross-section of the heart. Scale bar = 2000 μm; **B** The cardiomyocyte cross-section area (CSA) was stained with H&E staining. Scale bars = 50 μm; **C** quantification of cardiomyocyte cross-section areas. Data are expressed as the mean ± SD (n = 6 rats per group). *P < 0.05 relative to CTL group. ^†^*P* < 0.05 relative to Ang II group

As shown in Fig. 2, H&E staining of the cross-section of the heart showed that the thickness of the ventricular wall (Fig. 2A) and cardiomyocyte cross-sectional areas (CSA) (Fig. 2B) were significantly increased in the Ang II group (*P* < 0.01), and these increases were significantly alleviated by DAPA (*P* < 0.05) (Fig. 2C). These results also confirmed the protective role of DAPA in Ang II-induced cardiomyocyte hypertrophy.

### DAPA alleviated Ang II-induced myocardial fibrosis

**Fig. 3:**
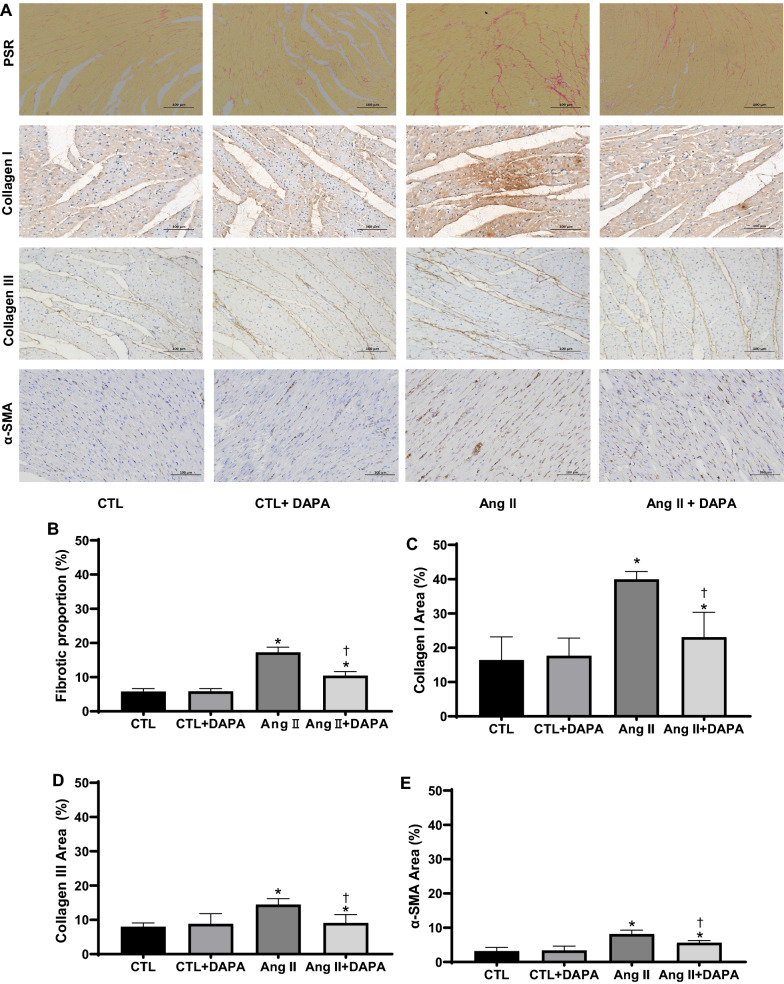

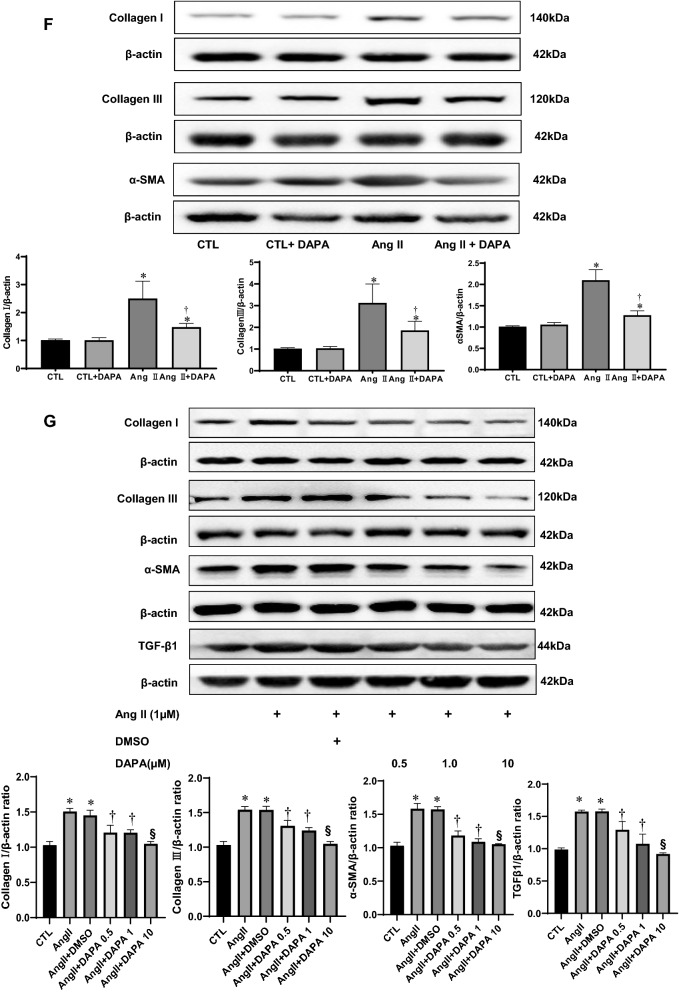
DAPA treatment suppresses matrix accumulation and myocardial fibrosis in vitro and in vivo. **A** The effects of DAPA on the fibrosis in myocardial tissue were observed by PSR staining (top), Scale bars = 100 μm; red color represents collagen fibers deposition. Meanwhile, the expression of type I collagen, type III collagen and α-SMA (bottom) in the myocardium was detected by immunostaining and DAPI staining. Scale bars = 100 μm; **B** quantification of red color in bar graph. OD values are presented as mean ± SD (n = 6 rats per group). **C**–**E** Immunohistochemical analysis for the effect of DAPA on Ang II-induced expression of type I collagen, type III collagen and α-SMA in myocardial tissue. Values are presented as mean ± SD (n = 6 rats per group). *P < 0.05 relative to CTL group. ^†^*P* < 0.05 relative to Ang II group. **F** Effects of DAPA treatment on expression of type I collagen, type III collagen, α-SMA in Ang II-infused rats were examined by immunoblotting. The relative ratio of type I collagen, type III collagen, α-SMA over β-actin was determined by densitometric analysis respectively.Values are means ± SD (n = 6). *P < 0.05 relative to CTL group. ^†^*P* < 0.05 relative to Ang II group. **G** DAPA inhibits Ang II-induced expression of type I collagen, type III collagen, α-SMA and TGF-β1 in CFs. CFs treated with the indicated concentrations of DAPA for 1 h were exposed to Ang II for 24 h. The relative ratio of type I collagen, type III collagen, α-SMA and TGF-β1 over β-actin was determined by densitometric analysis respectively.Values are means ± SD (n = 3). *DMSO* dimethyl sulfoxide. Values are means ± SD. *P < 0.05 relative to CTL group. ^†^*P* < 0.05 relative to Ang II group. ^§^*P* < 0.05 relative to Ang II plus DAPA 0.5 group

Picrosirius Red (PSR) staining showed that chronic Ang II infusion for 4 weeks significantly increased red-stained fibers in the shapes of bundles and sheets in the myocardial tissues of rats, DAPA treatment significantly lessened Ang Il-induced cardiac fibrosis. The collagen content in Ang II group was higher than in CTL group. However, DAPA treatment obviously attenuated the Ang II-induce increase of collagen (10.44 ± 1.19 vs. 17.27 ± 1.51, *P* < 0.01; Fig. 3A, B).

ECM synthesis, including collagen and fibronectin, plays an crucial role in myocardial fibrosis. To evaluate the effect of DAPA on collagen synthesis, immunohistochemistry and Western blotting were performed. Immunohistochemistry analysis demonstrated that α-SMA, type I and type III collagen were significantly increased in the myocardial tissue of the Ang II-infused rats. DAPA treatment markedly decreased the positive percentages of α-SMA, type I and type III collagen (Fig. 3A–E). Subsequently, Western blotting was performed to further confirm the above findings. As shown in Fig. 3F, Ang II also increased the expression of α-SMA, type I and type III collagen in the cardiac tissues, and these increases were significantly attenuated by DAPA. These results suggest that DAPA could effectively alliveated Ang II-induced myocardial fibrosis in rats.

In the adult heart, activated CFs also participate in the healing response after acute myocardial infarction and during chronic disease states characterized by augmented interstitial fibrosis and ventricular remodelling. A central cytokine involved in fibroblast activation, at least as defined in cultured fibroblasts, is transforming growth factor-β (TGF-β) [19, 20]. TGF-β and its downstream effectors constitute one of the most potent regulatory cascades for α-SMA gene expression and myofibroblast differentiation [21]. To further verify the protective effect of DAPA in vitro, CFs were pretreated with different concentrations of DAPA for 1 h, followed by incubation in 1 µM Ang II for 24 h. As shown in Fig. 3G, Ang II significantly increased the expression of α-SMA, TGFβ1, type I and type III collagen in CFs, which was significantly compromised by DAPA treatment in a dose-dependent manner.

### DAPA suppressed the activation of pro-fibrotic TGF-β1/Smad signaling in Ang II-infused rats

**Fig. 4:**
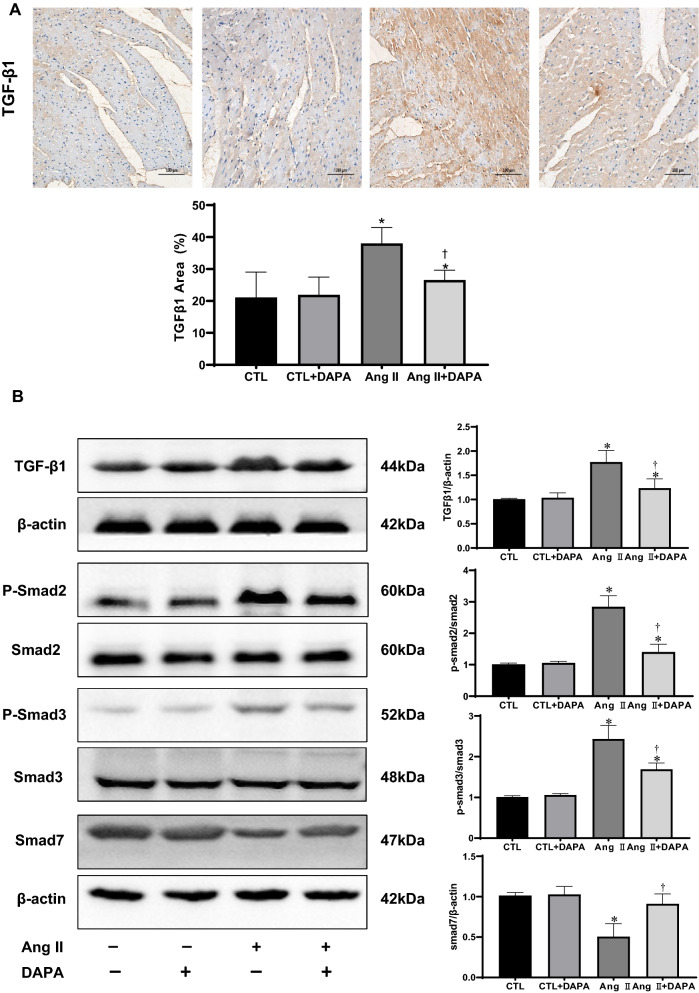
DAPA treatment inhibits the activation of the TGF-β1/Smad pathway in rats with continuous Ang II infusion. **A** Immunohistochemical analysis for the effect of DAPA on Ang II-induced expression of TGF-β1in myocardial tissue. Values are presented as means ± SD (n = 6 rats per group). *P < 0.05 relative to CTL group. ^†^*P* < 0.05 relative to Ang II group.Scale bars = 100 μm; **B** inhibition of Ang II infusion -induced activation of TGF-β1/Smad pathway. The relative ratio of TGF-β1 and Smad7 over β-actin was determined by densitometric analysis respectively. Also, the ratio of phosphor-Smad2/Smad2 and phosphor-Smad3/Smad3 was calculated based on densitometric analysis. Values are means ± SD (n = 6). *P < 0.05 relative to CTL group. ^†^*P* < 0.05 relative to Ang II group

The members of the TGF-β superfamily are critical regulators of remodeling and fibrosis. TGF-βs are released and activated in injured tissues, bind to their receptors and transduce signals in part through activation of cascades involving a family of intracellular effectors the receptor-activated Smads (R-Smads) [22]. We therefore investigated the effects of DAPA treatment on TGF-β/Smads signaling. As shown in Fig. 4A, immunohistochemical staining showed increased TGF-β1 expression in the Ang II-infused group. Compared with the Ang II group, DAPA markedly suppressed the expression of TGF-β1. Meanwhile, immunoblotting results demonstrated that TGF-β1 expression and the ratios of p-Smad2/Smad2 and p-Smad3/Smad3 experienced a significant up-regulation in response to Ang II. Whereas DAPA treatment significantly decreased Ang II-induced up-regulation of TGFβ1 levels and p-Smad2/Smad2 and p-Smad3/Smad3 ratios. Smad7, a negative inhibitor of TGF-β1/Smad signaling, was decreased in the Ang II group and greatly increased by DAPA treatment. These suggest that inhibiting TGF-β1/Smad pathway is one of the potential mechanisms for DAPA to prevent myocardial remodeling.

## Discussion

In the present study, we evaluated the effect of SGLT2i DAPA on Ang II-induced cardiac remodeling. In vivo, we found that DAPA treatment metigated Ang II-induced myocardial hypertrophy, fibrosis, and cardiac dysfunction without affecting serum glucose. In vitro, we confirmed that DAPA inhibited Ang II-induced collagen synthesis in CFs. Furthermore, we demonstrated an inhibitory role of DAPA on cardiac fibrosis by interfering with TGF-β1/Smads signaling cascades in myocardium. These results implied that DAPA can ameliorate cardiac remodeling in rats without diabetes, which provides evidence for the underway clinical trials and a basis for follow-up experiments.

DAPA is a newly oral antidiabetic drug of SGLT2i that enhances renal glucose excretion or glycosuria and reduces hyperglycemia [23, 24]. It is reported that lower risk of heart failure and death in patients initiated on SGLT2i versus other glucose-lowering drugs [25]. In Dapagliflozin and Prevention of Adverse Outcomes in Heart Failure (DAPA-HF) trial, patients who received DAPA had a significant 26% reduction in the risk of cardiovascular death or worsening HF compared to those who received placebo, with an 18% reduction in the risk of cardiovascular death and all­cause mortality [24]. These data indicating DAPA can exert cardiovascular protection beyond hypoglycemic effect. However, the precise impacts and mechanisms still need further exploration.

Chronic infusion of Ang II is a widely used method for establishing a model of cardiovascular disease [5]. To clarify the benefits of DAPA on myocardial remodeling, we used chronic Ang II-infused non-diabetic rat model in the study. As expected, DAPA significantly ameliorate cardiac function without lowering blood glucose. Previous results have indicated that continuous Ang II infusion at a high dose (1000 ng/kg/min) for 2 weeks increases LVEF% and LVFS%, while Ang II infusion for 4 weeks decreases LVEF% and LVFS% [17, 18]. Unlike these studies, a lower dose of Ang II perfusion for 4 weeks was used in our study. But, it still can be seen that Ang II-infusion increased LVEF% and LVFS% in rats. There may be two possible explanation for this, one is that Ang II induce myocardial hypertrophy leads to compensatory enhancement of myocardial contractility, the other is that conventional echocardiography can not detect the early early cardiac dysfunction.

QTVI takes high-frequency real-time images and is not affected by left ventricular preload and left atrial pressure, allowing detection of the early abnormalities of LV function [26]. While analyzing the QTVI results, we unexpectedly found that the decline of s, s′ and s_ave_ is earlier than LVEF in Ang II-infused rats (Table 3), further confirming that reduced systolic mitral annular peak velocity is a sensitive indicator of early subclinical systolic impairment. Early diastolic mitral annular motion velocities (e, e′ and e_ave_) are key indicators of active left ventricular myocardial relaxation, while changes in end-diastolic mitral annular motion velocities (a, a′ and a_ave_) are associated with left atrial contractility and left ventricular end-diastolic compliance [27, 28]. The ratio of early diastolic peak blood flow velocity (E) and e_ave_ (E/e_ave_) is positively associated with left ventricular filling pressure and indirectly reflects left ventricular diastolic function [29]. In our study, the decrease in e, e′, e_ave_, a, a′, a_ave_ and the increase in E/e_ave_ ratio in Ang II-infused group indicated a reduction in left ventricular diastolic function at the early stage of Ang II-induced rats. Interestingly, treatment of the rats with DAPA significantly improved the above parameters reflecting the LV systolic and diastolic function (Table 3).

Recent studies have suggested that speckle tracking echocardiography might be more sensitive than conventional echocardiography in detecting early cardiac function [30, 31]. To further evaluate the protective effect of DAPA in Ang II induced cardiac diyfunction, GLS of the left ventricle was detected. DAPA also significantly increased the GLS in Ang II rats (Fig. 1E and Table 3).

LV hypertrophy (LVH) and increased LV mass (LVM) are associated with an increased risk for heart failure and sudden cardiac death [32]. In DAPA-LVH trial (A randomized controlled trial of dapagliflozin on left ventricular hypertrophy in people with type two diabetes), DAPA treatment significantly reduced LVM in people with T2D and LVH. This reduction in LVM was accompanied by reductions in systolic BP, body weight, visceral and subcutaneous adipose tissue, insulin resistance, and hsCRP [33]. In our study, DAPA treatment markedly ameliorate HW, HW/TL and LVW/TL ratio, IVSd, LVPWd and cardiomyocyte cross-sectional area in Ang II-induced rats (Table 2 and Fig. 2). The amelioration of LVH suggests DAPA can initiate prevent remodelling and changes in left ventricular structure that may partly contribute to the cardio-protective effects of DAPA. Unlike previous studies, we failed to find a significant BP-lowering effect of DAPA. This may be related to the limitations of using tail-cuff method to measured BP in our study. Without animal familiarization tail-cuff measurements of blood pressure are most likely affected by acute responses to restraint stress. Some studies have found the antihypertensive effect of DAPA, which is believed to be related to weight reduction, modest diuretic effect, and potentially sodium depletion [34, 35]. Unfortunately, our research failed to give an explanation from these aspects.

Myocardial fibrosis, characterized by excess deposition of ECM and myofibroblast accumulation, is an integral feature of the remodelling of the failing heart [1, 36]. CFs play a paramount role in the repair and remodelling of the heart that occurs following myocardial infarction and pathological stress because of their exceptional plasticity to undergo conversions into myofibroblasts [37]. In this study, DAPA treatment for 4 weeks successfully alleviated the cardiac fibrosis, the deposition of type I and type III collagen, and α-SMA induced by Ang II infusion. In vitro, DAPA also inhibitedAng II-induced collagen synthesis in CFs (Fig. 3D). These are consistent with previous reports [11, 38–40]. Thus, inhibition of ECM synthesis and fibroblast-to-myofibroblast conversion may be the important mechanisms for DAPA to prevent myocardial fibrosis. Secreted TGF-β1 and activation of Smad-dependent pathway upon injury is the main inducer of ECM production and fibroblast-to-myofibroblast conversion [41, 42]. In our research, DAPA suppressed the expression of TGF-β1 and the ratios of p-Smad2/Smad2 and p-Smad3/Smad3 in Ang II-infused rats. In addition, Smad7, the negative regulator of the TGF-β1/Smad pathway, was significantly increased by DAPA treatment (Fig. 4). These results demonstrate an inhibitory role of DAPA of cardiac hypertrophy and fibrosis by interfering with TGFβ1/Smads signalling in the myocardium. Nevertheless, a detailed mechanism by which DAPA regulates TGF-β1/Smad signaling needs further elucidation.

Currently, SGLT2i has received considerable attention for its potential cardioprotective effects and related research is increasing. Most studies focused on pathological conditions such as diabetes [43]. Here, we explored the cardioprotective effect of SGLT2i in the Ang-II-induced hypertension, which has demonstrated the non-hypoglycemic anti-fibrotic effect.

## Conclusions

In conclusion, in this study, we showed that SGLT2 inhibitor DAPA pretreatment attenuates myocardial hypertrophy, fibrosis and LV dysfunction in Ang II-infused rat model via negative regulation of TGF-β1/Smad signaling.

## Supplementary Information

**Additional file 1: Table S1.** The effect of DAPA on blood pressure detected by cardiac hemodynamic monitoring and non-invasive tail-cuff system respectively in normal and Ang II-infused rats treated with vehicle or DAPA.

## Data Availability

All data and materials are available upon request.

## Abbreviations

SGLT2: Sodium–glucose cotransporter 2
TGF-β: Transforming growth factor-β
Glu: Glucose
INS: Insulin
TC: Total serum cholesterol
TG: Triglyceride
LDL: Low-density lipoprotein
HDL: High-density lipoprotein
SCr: Serum creatinine
ALT: Serum alanine aminotransferase
AST: Aspartate aminotransferase
LDH: Lactate dehydrogenase
CK: Creatine kinase
BW: Body weight
SBP: Systolic blood pressure
DBP: Diastolic blood pressure
HW: Heart weight
LVW: Left ventricle weight
TL: Tibia length
IVSd: End-diastole interventricular septum thickness
LVEDd: Left ventricular end-diastolic dimension
LVEDs: Left ventricular end-systolic dimension
LVEDV: Left ventricular end-diastolic volume
LVESV: Left ventricular end-systolic volume
LVPWd: Diastolic left ventricular posterior wall thickness
LVFS: Left ventricular fractional shortening
LVEF: The left ventricular ejection fraction
HR: Heart rate
s: Peak systolic mitral annular velocity at lateral side
s′: Peak systolic mitral annular velocity at septal side
s_ave_: Average value of the peak systolic mitral annular velocity
a: Late diastolic mitral annular velocity at lateral side
a′: Late diastolic mitral annular velocity at septal side
a_ave_: Average value of the late diastolic mitral annular velocity
e: Early diastolic mitral annular velocity at the lateral side
*e*′: Early diastolic mitral annular velocity at the septal side
e_ave_: Average value of the early diastolic mitral annular velocity
E: Peak velocities of diastolic early transmitral Doppler flow
GLS: Global longitudinal strain
SDS: Sodium dodecyl sulphate
PAGE: Polyacrylamide gel electrophoresis
SD: Standard deviation
