# Supplementary material for: Dapagliflozin: a sodium–glucose cotransporter 2 inhibitor, attenuates angiotensin II-induced cardiac fibrotic remodeling by regulating TGFβ1/Smad signaling
Source: Cardiovasc Diabetol. 2021 Jun 11;20:121. doi: 10.1186/s12933-021-01312-8 (PMC8196449; doi:10.1186/s12933-021-01312-8)
Supplement: Supplementary file 1 — Additional file 1: Table S1. The effect of DAPA on blood pressure detected by cardiac hemodynamic monitoring and non-invasive tail-cuff system respectively in normal and Ang II-infused rats treated with vehicle or DAPA. [file 12933_2021_1312_MOESM1_ESM.pdf]

Additional file 1

Table S1. The effect of DAPA on blood pressure detected by cardiac hemodynamic monitoring and non-invasive tail-cuff system respectively in normal and Ang II-infused rats treated with vehicle or DAPA.

	**CTL**	**CTL+DAPA**	**Ang II**	**Ang II+DAPA**
SBP (mmHg)	130.6±8.5	131.4±7.4	214.8±9.6*	208.6±8.2*
DBP (mmHg)	105.6±6.8	106.7±8.2	185.02±8.4*	180.1±7.8*
MAP (mm)	113.4±5.8	114.2±8.6	190.3±9.4*	186.8±8.9*
HR	369±20	367±17	402±24*	408±21*

Values are means±SD. MAP were measured by the Millar pressure-volume conductance catheter system. SBP and DBP were monitored with non-invasive tail-cuff system. HR, heart rate; MAP, mean arterial pressure; SBP, systolic blood pressure; DBP, diastolic blood pressure. *P <0.05 relative to CTL group.
